# Recycled Components in Mantle Plumes Deduced From Variations in Halogens (Cl, Br, and I), Trace Elements, and ^3^He/^4^He Along the Hawaiian‐Emperor Seamount Chain

**DOI:** 10.1029/2018GC007959

**Published:** 2019-01-14

**Authors:** Michael W. Broadley, Hirochika Sumino, David W. Graham, Ray Burgess, Chris J. Ballentine

**Affiliations:** ^1^ School of Earth and Environmental Sciences University of Manchester Manchester UK; ^2^ Centre de Recherches Pétrographiques et Géochimiques Vandoeuvre‐Lès‐Nancy France; ^3^ Department of Basic Science, Graduate School of Arts and Sciences The University of Tokyo Tokyo Japan; ^4^ College of Earth, Ocean, and Atmospheric Sciences Oregon State University Corvallis OR USA; ^5^ Department of Earth Sciences University of Oxford Oxford UK

**Keywords:** volatile recycling, mantle evolution, noble gas, halogens

## Abstract

Halogens are primarily located within surface reservoirs of the Earth; as such they have proven to be effective tracers for the identification of subducted volatiles within the mantle. Subducting lithologies exhibit a wide variety of halogen compositions, yet the mantle maintains a fairly uniform signature, suggesting halogens may be homogenized during subduction to the mantle or during eruption. Here we present halogen (Cl, Br, and I), K, noble gas, and major and trace element data on olivines from three seamounts along the Hawaiian‐Emperor seamount chain to determine if the deep mantle source has retained evidence of halogen heterogeneities introduced through subduction. High Ni contents indicate that the Hawaiian‐Emperor mantle source contains a recycled oceanic crust component in the form of pyroxenite, which increases from the 46% in the oldest (Detroit) to 70% in the younger seamount (Koko). Detroit seamount retains mid‐ocean ridge basalts (MORB)‐like Br/Cl and I/Cl, while the Br/Cl and I/Cl of Suiko and Koko seamounts are higher than MORB and similar to altered oceanic crust and dehydrated serpentinite. Helium isotopes show a similar evolution, from MORB‐like values at Detroit seamount toward higher values at Suiko and Koko seamounts. The correlation between pyroxenite contributions, Br/Cl, I/Cl, and ^3^He/^4^He indicates that subducted material has been incorporated into the primordial undegassed Hawaiian mantle plume source. The identification of recycled oceanic crustal signatures in both the trace elements and halogens indicates that subduction and dehydration of altered oceanic crust may exert control on the cycling of volatile elements to the deep mantle.

## Key Points

- Trace elements in Emperor seamount olivines indicate magmatism sampled an increasing proportion of pyroxenite through time
- Higher than mantle Br/Cl and I/Cl in the younger seamounts indicate that the pyroxenite contains a subducted volatile component
- Helium isotope variations indicate that Hawaiian plume magmatism also sampled primitive material derived from the deep mantle

## 1 Introduction

The wide range of differing geochemical signatures within submarine basalts is considered indicative of the heterogeneous nature of the Earth's mantle (Hofmann, 2003; Kurz et al., 1982; Mukhopadhyay, 2012; White, 2010). Variable and high ^3^He/^4^He ratios in intraplate ocean island basalts (OIB = 5–50 R_A_, where R_A_ is the ^3^He/^4^He of atmosphere = 1.39 × 10^−6^) compared to lower, relatively uniform ratios of mid‐ocean ridge basalts (MORB = 8 ± 1 R_A_) provide fundamental evidence for at least two mantle reservoirs within the Earth (e.g., Graham, 2002; Stuart et al., 2003). The high ^3^He/^4^He OIB are believed to tap into the primordial, less degassed mantle reservoir through rising mantle plumes, while MORB are derived from the passive upwelling of the depleted and degassed convecting upper mantle (Allègre et al., 1983; Honda et al., 1993; Mukhopadhyay, 2012). Although OIB are often characterized by their high ^3^He/^4^He ratios, there is significant geochemical variation between different ocean islands and even within the same island chain, indicating that the mantle source is heterogeneous over relatively small length scales (Huang et al., 2011; Jackson et al., 2014; Weis et al., 2011).

The heterogeneity within different OIB sources is in part attributed to varying amounts of oceanic lithosphere and crustal material introduced to the mantle through subduction (Hofmann & White, 1982). Ocean islands that are believed to sample the remnants of subducted oceanic crust (e.g., Canary Islands, St Helena, Azores etc.) are characterized by lower ^3^He/^4^He and ^143^Nd/^144^Nd, and higher radiogenic ^87^Sr/^86^Sr and ^206^Pb/^204^Pb signatures, than the primordial‐type plumes (e.g., Hawaii and Iceland, Eisele et al., 2002; Hanyu et al., 2011). This is due to the enrichment in incompatible elements such as U, Th, Rb, and Sr within crustal lithologies, which when subducted and isolated for >1 Ga develop a distinct radiogenic signature within the mantle (Day et al., 2010; Stracke et al., 2003). Although plumes derived from a subducted source should have a ^3^He/^4^He ratio lower than primordial plumes, there are some plumes (Pitcairn, Society, Samoa) that are able to retain a high ^3^He/^4^He ratio attributed to the primordial mantle reservoir and yet trend toward more isotopically enriched Sr, Nd, and Pb signatures than other hot spots (Graham et al., 1990; Honda & Woodhead, 2005; Jackson et al., 2007, 2014). This suggests that OIB's can sample both primordial and recycled components that have remained separated long enough to retain their distinct geochemical signatures (White, 1985).

The discovery and prevalence of recycled atmospheric/seawater noble gases within both MORB and OIB mantle sources indicates that a small fraction of noble gases can survive the subduction process and become widely distributed throughout the mantle (Holland & Ballentine, 2006; Parai & Mukhopadhyay, 2015). Furthermore, evidence from halogens within a mantle wedge peridotite, arc setting xenoliths, and within back‐arc basin basalts suggests that marine volatiles from slab‐derived sedimentary pore fluids, serpentinites, and seawater can be subducted into the upper mantle (Broadley et al., 2016; Kendrick, Arculus, et al., 2014; Kendrick et al., 2017; Kobayashi et al., 2017; Sumino et al., 2010). The mechanisms through which surface volatiles are able to survive subduction are not well known. However, noble gases and halogens can be sited within the lattice of hydrous minerals such as amphibole and serpentine, which are capable of delivering volatiles to at least 100 km (Jackson et al., 2013; Kendrick, Honda, et al., 2013; Kobayashi et al., 2017; Sumino et al., 2010).

While the halogen elemental ratios measured within OIB samples may show variations between different islands, they generally have the same Br/Cl and I/Cl composition as MORB samples (Kendrick et al., 2012; Kendrick, Jackson, et al., 2014, 2015). The uniformity of the Br/Cl and I/Cl ratios within the mantle, including OIB that have radiogenic isotopic signatures indicative of subducted oceanic crust and sediments, is at odds with the wide range of halogen compositions within subducting lithologies (Chavrit et al., 2016; Kendrick et al., 2011). The relatively homogeneous halogen signature of MORB and OIB samples has been suggested to result from subduction of multiple lithologies that average to a mantle‐like halogen composition, and therefore, subduction does not introduce any major halogen heterogeneity to the mantle (Kendrick et al., 2018). This would require the halogens to be decoupled from other volatile species such as oxygen, sulfur, and nitrogen, which not only exhibit heterogeneity in the mantle but have also been used to trace the subduction of specific lithologies (Cabral et al., 2013; Sano et al., 1998; Thirwall et al., 1997). The full extent of halogen recycling and mixing within the mantle is therefore still not well understood, and whether subduction can introduce halogen heterogeneities to the mantle remains to be established.

The significant geochemical heterogeneity of oceanic hot spot lavas can provide means for evaluating the different volatile components within the mantle and how the contribution of these components can vary through time. The Hawaiian‐Emperor seamount chain, the physical manifestation of the Hawaiian Plume, can be traced back to at least 76 Ma (Duncan & Keller, 2004). The Hawaiian mantle source shows considerable geochemical heterogeneity, with radiogenic isotopes indicating the presence of at least two potentially distinct mantle end‐members defined as the Loa and Kea components (Eiler et al., 1996; Weis et al., 2011). The Loa component is defined by its low ^143^Nd/^144^Nd and ^206^Pb/^204^Pb, and high ^87^Sr/^86^Sr. It is considered to contain the remnants of subducted oceanic crust including sediments (Blichert‐Toft et al., 1999; Huang et al., 2011; Hauri, 1996). The Kea component in contrast has high ^143^Nd/^144^Nd and ^206^Pb/^204^Pb and low ^87^Sr/^86^Sr and has been suggested to represent the incorporation of MORB‐source mantle, oceanic lithosphere, and young recycled oceanic crust into the plume (Eiler et al., 1996; Hanyu et al., 2010; Hauri, 1996). These two components have been geographically separated into the northern Kea trend and the southern Loa trend for the last 5 Ma of Hawaiian volcanism (Huang et al., 2011; Tanaka et al., 2008; Weis et al., 2011).

Recycled oceanic crust in the mantle can exist as pyroxenite or eclogite after having undergone metamorphism during subduction. Melts produced from recycled crust within a mantle source are expected to crystallize olivines with higher Ni, and lower Ca and Mn compared to melts originating from a peridotite mantle source (Herzberg, 2010; Sobolev et al., 2005, 2007). The current Hawaiian mantle source is modeled to contain 15–20% pyroxenite from the subduction of oceanic crust, indicating that the chemical makeup of subducting lithologies can be preserved in the mantle together with unique isotopic signatures (Sobolev et al., 2007)

While the Emperor seamounts are similar to the Kea‐type component of the Hawaiian Islands, they still show geochemical heterogeneity, with a temporal evolution from more MORB‐like ^87^Sr/^86^Sr and ^3^He/^4^He in the older seamounts to values similar to the modern Kea trend of the Hawaiian Islands (Keller et al., 2000; Keller et al., 2004; Regelous et al., 2003). ^3^He/^4^He signatures ranging from 10 to 24 R_A_ have been measured by crushing of olivine phenocrysts from the Emperor seamounts, indicating that olivine can retain mantle‐derived volatiles within its fluid and melt inclusions over periods of millions of years (Keller et al., 2004). The Hawaiian‐Emperor sample suite therefore shows a temporal trend, with increasing influence from deeper mantle‐derived components including the potential incorporation of subducted oceanic crust within the younger volcanics.

Trace element analysis of Ni, Ca, Cr, and Mn in olivine phenocrysts can provide unique insight into the potential contribution of recycled pyroxenite along the Hawaiian‐Emperor seamount chain (Sobolev et al., 2005, 2007). Variations in the amount of recycled pyroxenite contributing to the formation of the Emperor seamount chain may also be evident in other volatile elements such as halogens that are thought to be readily recycled back to the mantle (Broadley et al., 2016; Sumino et al., 2010). Evidence for the retention of mantle‐derived He within Emperor seamount olivine phenocrysts suggests that they have been able to resist complete overprinting from the surrounding marine environment (Keller et al., 2004). The analysis of halogen and noble gas analysis in the same olivine samples provides means to assess whether halogens can also be retained over millions of years and gives insight into volatile recycling in the mantle through time. We present a combined halogen and noble gas study of olivine‐hosted fluid inclusions, as well as bulk olivine separates, together with trace element analysis within several of the Emperor seamounts, to gain further understanding of the nature of subducted components within the primitive Hawaiian mantle source.

## 2 Samples and Analytical Methods

### 2.1 Emperor Seamount Samples

Samples were obtained from tholeiitic picrite lava flows from the shield‐building stage of three Cretaceous seamounts along the Emperor seamount chain. At sufficiently low pressures (<1 MPa; Edmonds et al., 2009) halogens can degas into the vapor phases and potentially be fractionated. In order to limit the degassing of both the halogens and noble gases, samples were chosen from submarine lava flows (Dalrymple et al., 1980; Duncan and Huang et al., 2005; Keller et al., 2004).

Samples were collected from seamounts that span the length of the observable volcanic chain. This was done in order to observe any temporal variations of noble gas and halogen compositions in the Hawaiian Plume source. Samples from the following three seamounts were analyzed: Detroit seamount (76 Ma), Suiko seamount (65 Ma), and Koko seamount (49 Ma, Tarduno et al., 2003). Samples originating from Detroit and Koko were recovered as part of Leg 197 of the Ocean Drilling Program (ODP) from sites 1203 and 1206, respectively (Tarduno et al., 2003). Samples from Suiko seamount were collected at site 433 during Leg 55 of the Deep Sea Drilling Project (Dalrymple et al., 1980). The majority of the olivine samples used for this study were obtained from existing material used previously for ^3^He/^4^He determinations (Keller et al., 2004). Additional olivine samples from the same lava flows were prepared to ensure sufficient material was available for both halogen and noble gas analysis. These additional olivine separates were obtained by selecting phenocryst‐rich sections of the lava flow from the International Ocean Drilling Programs core repository.

**Figure 1:**
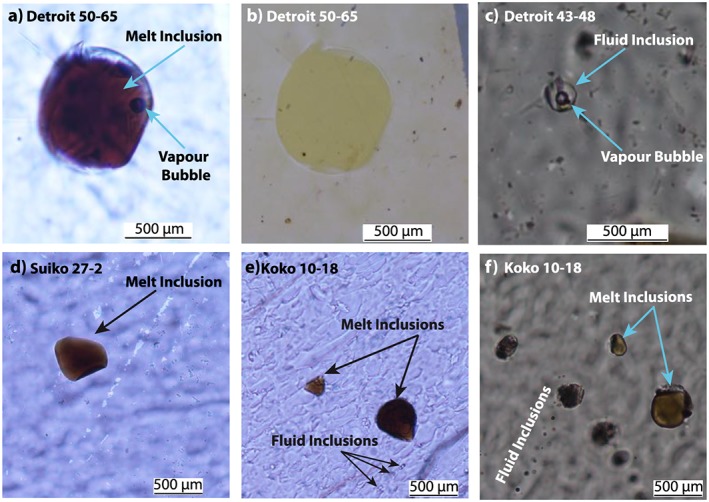
Photomicrograph images of melt and fluid inclusions in the Emperor seamount olivine phenocrysts. A glass melt inclusion containing a vapor bubble is shown in Detroit samples 50‐56 under transmitted (a) and reflected (b) light. Vapor‐dominated fluid inclusions occur in conjunction with melt inclusions in all samples but are significantly less abundant. Fluid inclusions are randomly distributed or occur in small groupings with no evidence of inclusions trails crossing grain boundaries, suggesting that they are a primary in nature. An example of a fluid inclusion and its associated vapor bubble is shown in Detroit samples 43‐48 (c). Examples of melt inclusions are shown for sample Koko 10‐18 (d) and Suiko 27‐2 (e) and grouped melt and fluid inclusions in Koko 10‐18 (f).

The tholeiitic picrite lavas sampled are rich in olivine phenocrysts, containing between 20 and 30 vol % olivine grains of up to 3 mm in size. The olivine is relatively unaltered containing pristine melt inclusions and rare vapor‐rich fluid inclusion (Figure 1). Some of the olivine samples show instances of limited alteration to iddingsite, carbonate, and clay minerals around the mineral rims (Figure S1 in the supporting information). Olivine phenocrysts are present within a tholeiitic groundmass containing in varying quantities: plagioclase, clinopyroxene, titanomagnetite, and devitrified glass (Huang et al., 2005).

### 2.2 Mineral Chemistry

Major and trace element compositions of the olivine phenocrysts from four polished thin sections were analyzed using a Cameca SX 100 electron microprobe at the University of Manchester. A total of 350 spot measurements were taken from 66 olivine phenocrysts. All analyses were performed using a beam current of 20 nA and an accelerating voltage of 15 kV. Counting times for all elements (Si, Al, Cr, Fe, Mn, Mg, Ca, Na, K, Ni and Co) was 60 s, determined as the optimal configuration to obtain the best detection limits. Prior to analysis the background and sensitivity of the microprobe was determined using well‐characterized silicate, oxide, and metal standards (Gregory et al., 2017).

### 2.3 Halogen Analysis

Olivine grains from core sections were liberated from the surrounding groundmass by coarsely crushing the basalt. The resulting crushed fraction was then sieved and handpicked under a binocular microscope to separate the olivine from the surrounding groundmass. Olivine crystals were cleaned ultrasonically in deionized water for 20 min, followed by 10 min in 1‐N nitric acid to remove any adhered groundmass and then a further 5 min in acetone as a final cleaning step. The samples were then handpicked under a binocular microscope a final time to ensure only the least altered olivine grains without adhering groundmass were selected for analysis.

Measurement of the halogens was conducted using neutron irradiation—noble gas mass spectrometry and methods described previously (Broadley et al., 2016; Ruzié‐Hamilton et al., 2016). Samples weighing ~50 mg were wrapped in Al foil and placed into sealed evacuated silica glass tubes along with a hornblende Hb3gr flux monitor (mean *J* value of 0.006288 ± 0.000028, Turner et al., 1971). The production of noble gases proxy isotopes (^38^Ar_Cl_, ^80^Kr_Br_, and ^128^Xe_I_) formed during irradiation was monitored using several scapolite and Shallowater meteorite I‐Xe standards (Johnson et al., 2000; Kendrick, 2012; Ruzié‐Hamilton et al., 2016). Samples were irradiated for a total of 24 hr at the Petten reactor, The Netherlands. The samples were exposed to a fast neutron flux of 2 × 10^18^ neutrons/cm^2^, a thermal flux of 5 × 10^18^, and a resonance flux of 1 × 10^17^ neutrons/cm^2^ as determined from monitor minerals.

Irradiated samples were loaded into the crushing line, comprising modified Nupro® hand‐operated valves (Stuart & Turner, 1992) in order to release noble gases from fluid inclusions within the samples. Each sample was crushed in a single step with a total of 20 crushes in order to ensure maximum gas release. Released gases were then purified on a SAES® NP10 Al‐Zr getter at 250 °C for 5 min before being analyzed using the MS1 mass spectrometer, University of Manchester (Broadley et al., 2016). Crushed powders were then weighed, repackaged in Al foil, and loaded into a tantalum resistance furnace for heating. Samples were heated in two steps; one at 1400 °C that releases the majority of the gas held within the samples and another at 1600 °C to remove any remaining gas and prepare the furnace for the next sample. Gases released during step heating were purified and analyzed using the same procedure as outlined above for the crushing analysis.

### 2.4 Noble Gas Analysis

Helium, neon, and argon isotopes were determined on 0.8–1.3 g of unirradiated olivine crystals. For the Detroit samples, both light and dark olivines were analyzed together, as there was not sufficient material for the two groups of olivines to be analyzed separately. Previous analysis of the light and dark olivines showed no systematic difference in He isotopic ratios therefore justifying this approach (Keller et al., 2004). The noble gases were released from the olivines via crushing using a magnetic solenoid crusher (Sumino et al., 2001). Each sample was crushed an average of 4 times (100, 500, 1000, and 2000 strokes) to ensure efficient gas release from any fluid inclusions. Prior to analysis, samples were baked overnight at 200 °C to reduce adsorbed atmospheric contamination. Extracted noble gases, purified by a series of Ti‐Zr getters, were adsorbed on to a cryogenically cooled trap containing sintered‐stainless steel with temperature‐controlled selective desorption of individual noble gases before isotopic analysis on a modified VG5400 mass spectrometer at the University of Tokyo, following the procedure described in Sumino et al. (2001).

## 3 Results

### 3.1 Olivine Chemistry

Average major and trace element data from multiple electron probe microanalysis (EPMA) measurements of olivine from each seamount are presented in Table 1, with the full EPMA data set available in the supporting information. Olivines from the seamounts show a range of forsterite (Fo = Mg/Mg + Fe) numbers, which successively decrease with age, from an average Fo of 87 (± 4.5, standard deviation) in olivines from Detroit to a lower average value of 86 (± 1.3) and 82 (± 1.8) in Suiko and Koko, respectively. Olivine from Suiko seamount ranges between Fo_81.7–87.4_, while those from the Koko seamount range between Fo_76.5–85.5_. Detroit seamount has two distinct olivine populations within the lava flows, which are distinguished by their light and dark coloring. Dark olivine has lower Fo numbers between Fo_73.9–78.0_ when compared to the lighter olivine Fo_75.2–90.0_. These values are similar to measurements previously reported on the same samples (Keller et al., 2004).

**Table 1:** Average Major and Trace Element Composition (in Oxide wt % and ppm) of Olivines From EPMA Data

Sample	SiO_2_	Al_2_O_3_	FeO	MgO	Cr (ppm)	Mn (ppm)	Ca (ppm)	Ni (ppm)	Co (ppm)	Total	Fo#
Detroit 100‐105											
99 analyses	39.9 ± 0.9	0.06 ± 0.02	12.6 ± 3.2	46.7 ± 2.6	453 ± 181	1366 ± 351	1938 ± 148	2441 ± 622	245 ± 42	100.1	86.8 ± 3.3
Detroit 100‐05 Dark											
5 analyses	38.0 ± 0.3	0.03 ± 0.01	20.8 ± 0.3	40.2 ± 0.1	129 ± 9	2480 ± 291	2244 ± 44	1019 ± 111	249 ± 25	99.8	77.5 ± 0.3
Detroit 50‐65											
48 analyses	39.6 ± 0.1	0.07 ± 0.01	13.7 ± 0.8	45.8 ± 0.8	544 ± 194	1465 ± 468	1970 ± 298	2365 ± 806	251 ± 42	99.9	85.5 ± 1.1
Detroit 50‐65 Dark											
4 analyses	37.5 ± 0.4	0.03 ± 0.01	25.0 ± 3.0	36.5 ± 2.3	92^a^	2828 ± 151	2290 ± 85	705 ± 108	n.d	99.9	72.3 ± 6.0
Suiko											
99 analyses	39.5 ± 0.5	0.05 ± 0.01	13.5 ± 1.2	45.62 ± 1.1	463 ± 102	1409 ± 210	1805 ± 91	2765 ± 299	249 ± 45	99.5	85.8 ± 1.3
Koko											
99 analyses	38.5 ± 0.5	0.03 ± 0.01	16.1 ± 1.6	43.6 ± 1.3	306 ± 60	1605 ± 220	1815 ± 87	2355 ± 233	256 ± 71	99.1	82.8 ± 1.8

*Note*. Major and trace elemental composition are determined on electron microprobe. Data represent an average of multiple analyses on thin sections from each seamount. Ni, Cr, Mn, Ca, and Co were measured at higher precision and are reported in parts per million, Fo# = molar Mg/(Mg + Fe). EPMA = electron probe microanalysis.

a Single analysis and therefore standard deviation could not be determined. n.d = not determined.

**Figure 2:**
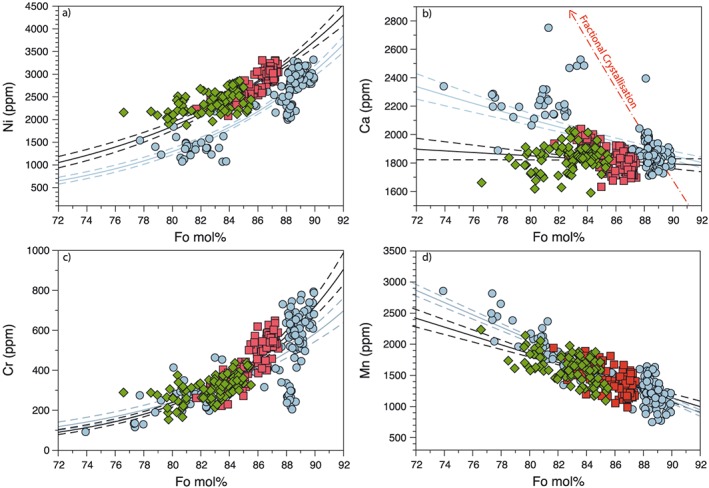
(a–d) Major and trace element composition of individual EPMA measurements from olivine phenocrysts for each of the Emperor seamounts analyzed in this study. Concentrations are displayed in ppm plotted against molar percentage of forsterite. Distinct compositional trends are shown by solid (least squares average) and dashed (95% confidence interval) lines for Detroit (blue) and a combination of Suiko and Koko (black). The younger seamounts (Suiko and Koko) are enriched in Ni (a) and depleted in Ca (b) relative to the older Detroit seamount. Distinct compositional differences between Detroit and Suiko/Koko indicate there has been a compositional change within the Hawaiian hot spot between the eruption of Detroit and Suiko. Calcium does not follow the expected path of fractional crystallization, as shown by the dashed red line. The fractional crystallization line was calculated using Petrolog software (Danyushevsky & Plechov, 2011), assuming a fertile peridotite melt at 3 GPa and 1515 °C (Walter, 1998). EPMA = electron probe microanalysis.

There is a correlation between the Ni, Mn, and Cr concentrations within the olivines and the Fo number with all three seamounts generally following fractional crystallization profiles as shown by the regression lines in Figure 2. While Ca concentrations also appear correlated with Fo numbers in the Detroit olivines, the data for Suiko and Koko do not plot along an expected fractional crystallization line (Figure 2b). This may indicate that Ca within the Suiko and Koko olivines has experienced zoning and disequilibrium diffusion, leading to lower Ca concentrations at a given Fo number (Gavrilenko et al., 2016). This will preferentially affect Ca given its lower diffusion coefficient in olivine relative to Mg, Fe, Ni, Cr, and Mn. During crystallization, the faster diffusing elements will be able to reequilibrate across any zoning feature while Ca will remain only partially equilibrated and plot off a normal liquid line of descent (Figure 2b; Gavrilenko et al., 2016)

The Emperor seamount olivines define two compositional trends shown in Figure 2, with Koko and Suiko falling on the same trend of element versus Fo composition, while Detroit olivines appear to form separate trends for Ni and Ca (Figures 2a and 2b). At a given Fo content the olivine Ni content is higher within samples originating from Koko and Suiko seamounts when compared to Detroit. For example, at Fo values ≤85 the maximum measured Ni concentration within Koko is 2,855 ± 137 ppm, which is greater than both Suiko (2647 ± 134 ppm) and Detroit (2451 ± 131 ppm) (Figure 2a).

The Ca concentration within Suiko and Koko olivines is similar and less sensitive to changing Fo number compared to Detroit. The range of Ca concentrations within Suiko (1,632–2,039 ppm) and Koko (1,590–2,027 ppm) overlaps and is lower than the concentrations within Detroit olivines (1,706–2,752 ppm). Variations in Mn are less pronounced than for Ca but the trend is similar with lower Mn concentrations within Suiko and Koko compared to Detroit at Fo numbers lower than 86 (Figure 2d).

### 3.2 Halogens

Concentrations of halogens and potassium obtained through crushing and step heating are presented in Table 2. The concentration of halogens within the olivine is low, with the range of Cl (0.2–8.1 ppm), Br (0.4–36.2 ppb), and I (0.01–0.8 ppb) being between 2 and 3 orders of magnitude lower than that of OIB basaltic glass (Kendrick, Jackson, et al., 2014). Between 6 and 51% of the total halogen abundance are released during the crushing step (Table 2) and are assumed to be sited in fluid inclusions due to their incompatibility within silicate minerals (Schilling et al., 1980). Crushing and step heating can be used to help distinguish between potentially different volatile components trapped within the fluid inclusions, and melt inclusions and the mineral matrix, respectively. Previous measurements of He released from crushing of these samples suggest that the samples contain fluid inclusions or vapor bubbles associated with melt inclusions. Petrographic evidence suggests both fluid and melt inclusions are present within the samples, although both are very rare (supporting information). The majority of the halogens within the Emperor seamount olivines (typically >70% total halogen release) are released during step heating suggesting the halogens may be predominantly sited within melt inclusions of the olivine. Relative concentrations of Cl and Br are similar across all three seamounts. However, there is an increase in average I concentrations from Detroit (0.12 ppb) toward the younger seamounts of Suiko (0.29 ppb) and Koko (0.32 ppb).

**Table 2:** Halogen Concentrations and Ratios From Emperor Seamount Olivines

Sample	Weight (g)	Method	Cl (ppm)	±	Br (ppb)	±	I (ppb)	±	K (ppm)	±	Br/Cl (×10^−3^)	± (×10^−3^)	I/Cl (×10^−5^)	± (×10^−5^)	K/Cl	±
Detroit																
Detroit 43‐48	0.0544	20 Crushes	1.20	0.16	3.62	0.04	0.120	0.015	16.2	0.4	3.02	0.41	9.59	1.83	13.54	1.85
	0.0184	1400 °C	1.36	0.07	5.54	0.09	0.160	0.022	13.1	0.6	4.08	0.20	11.92	1.68	9.67	0.65
Detroit 50‐65	0.0564	20 Crushes	0.28	0.01	1.39	0.03	0.040	0.005	2.1	0.1	4.89	0.25	12.49	1.79	7.29	0.54
	0.0166	1400 °C	2.97	0.06	11.77	0.12	0.320	0.018	34.4	0.3	3.97	0.09	10.77	0.64	11.60	0.25
Detroit 100‐105	0.0575	20 Crushes	2.27	0.03	10.70	0.14	0.070	0.009	2.6	0.1	4.73	0.09	3.11	0.39	1.15	0.04
	0.0275	1400 °C	2.21	0.03	6.04	0.07	0.110	0.008	24.0	0.3	2.75	0.05	5.19	0.39	10.89	0.21
Detroit 43‐48 Dark	0.0552	20 Crushes	0.16	0.02	0.89	0.02	0.040	0.007	1.0	0.1	5.70	0.61	28.35	5.37	6.30	0.72
	0.0254	1400 °C	0.66	0.05	2.14	0.06	0.070	0.015	13.0	0.5	3.25	0.27	10.77	2.40	19.72	1.71
Detroit 50‐65 Dark	0.048	20 Crushes	0.23	0.03	0.57	0.05	0.030	0.003	2.7	0.1	2.48	0.38	15.11	2.40	11.68	1.54
	0.0246	1400 °C	1.65	0.05	5.30	0.11	0.280	0.015	37.8	0.5	3.22	0.11	17.29	1.04	22.93	0.71
Detroit 100‐105 Dark	0.0443	20 Crushes	2.07	0.03	11.23	0.12	0.070	0.007	2.2	0.1	5.45	0.09	3.58	0.32	1.07	0.02
	0.0205	1400 °C	3.93	0.09	9.80	0.09	0.090	0.001	38.3	0.4	2.50	0.07	2.40	0.29	9.75	0.25
Suiko																
Suiko 27‐2	0.0485	20 Crushes	0.55	0.02	2.64	0.03	0.078	0.008	9.1	0.4	4.78	0.16	14.03	1.54	16.54	0.88
	0.008	1400 °C	8.14	0.14	36.18	0.34	0.823	0.040	145.9	1.9	4.44	0.09	10.13	0.54	17.95	0.38
Suiko 24‐7	0.0586	20 Crushes	0.06	0.01	0.41	0.01	0.020	0.002	5.1	0.1	7.14	1.58	34.62	8.02	89.80	18.79
	0.0181	1400 °C	0.74	0.10	3.29	0.08	0.259	0.097	60.1	0.6	4.44	0.63	34.94	14.00	81.05	11.31
Koko																
Koko 13‐16	0.0462	20 Crushes	0.16	0.01	0.73	0.01	0.035	0.003	3.8	0.2	4.49	0.38	21.76	2.36	23.33	2.26
	0.0184	1400 °C	1.28	0.03	4.50	0.14	0.600	0.048	32.4	0.4	3.52	0.14	46.89	3.97	25.36	0.72
Koko 10‐18	0.0478	20 Crushes	0.14	0.08	0.83	0.02	n.d	n.d	1.7	0.2	5.79	3.02	n.d	n.d	12.13	6.47
	0.0173	1400 °C	1.85	0.04	16.26	1.32	0.322	0.046	46.1	0.4	8.81	0.74	17.43	2.51	24.98	0.52

*Note*. n.d = not determined. Analytical uncertainties are 1*σ*.

**Figure 3:**
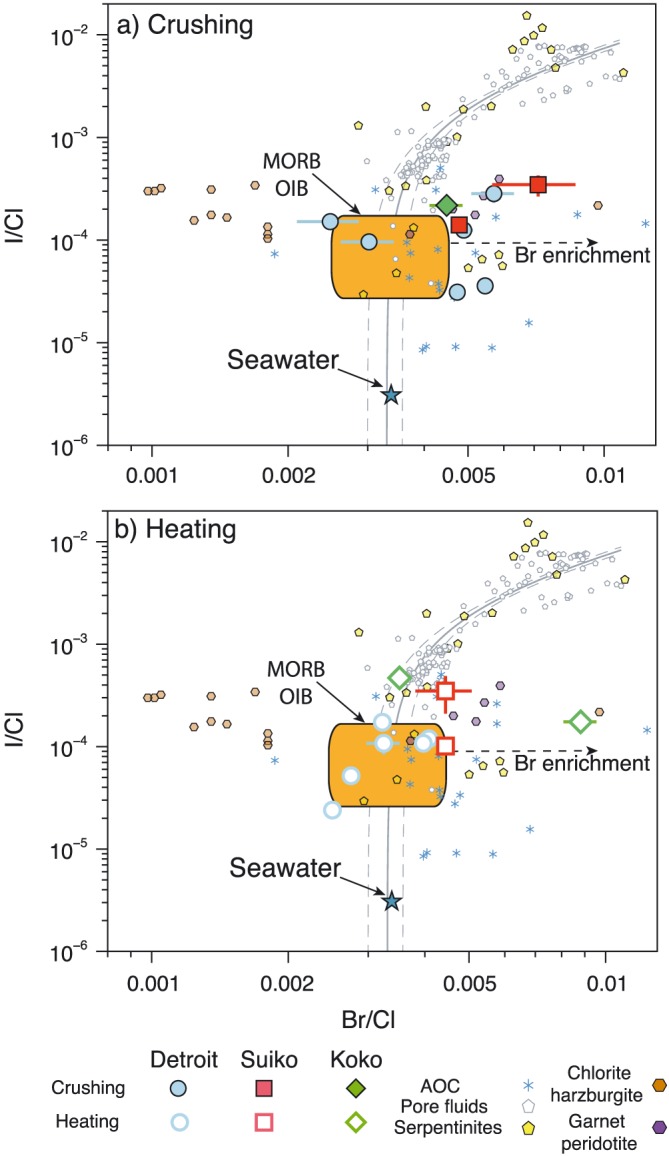
Log‐log plot of Br/Cl versus I/Cl weight ratios from data obtained from crushing (a) and step heating (b). Olivines from each of the Emperor seamounts analyzed in this study are plotted with seawater, MORB/OIB (Kendrick et al., 2012; Kendrick, Jackson, et al., 2014) marine pore fluids/brines are shown by solid (least squares average) and dashed (95% confidence interval) gray lines (Kastner et al., 1990; Martin et al., 1993; Muramatsu et al., 2007), altered oceanic crust (AOC) fluids (Chavrit et al., 2016), serpentinites (Kendrick, Honda, et al., 2013), chlorite harzburgite, and garnet peridotites (Kendrick et al., 2018). Data obtained from crushing release show the Emperor seamounts have enriched Br/Cl ratios compared to the MORB and OIB but similar to AOC fluids and garnet peridotites. Data from heating steps show different ratios for each seamount, with Detroit having mantle‐like Br/Cl and I/Cl while samples from Suiko and Koko are enriched in Br and I and have Br/Cl and I/Cl greater than the range of mantle values. MORB = mid‐ocean ridge basalts; OIB = ocean island basalts.

**Figure 4:**
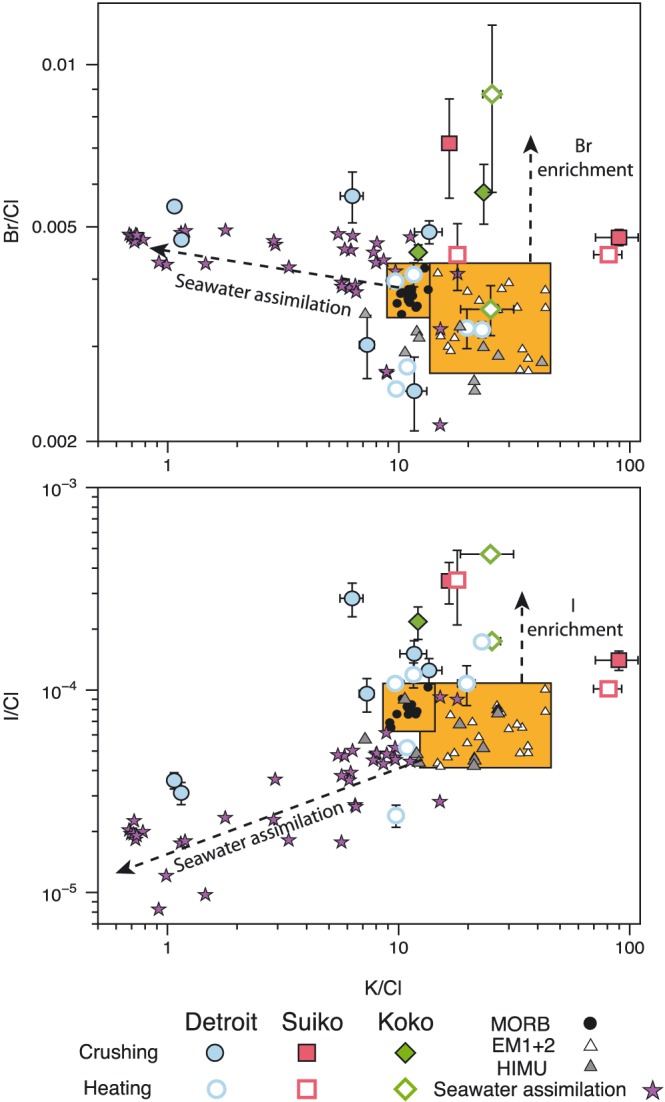
Log‐log plot of Br/Cl (a) and I/Cl (b) versus K/Cl weight ratios of the Emperor seamounts from both crushing and step heating. Olivines from the Emperor seamounts are plotted together with data from Pacific MORB, EM1, EM2, and HIMU and samples exhibiting evidence of seawater assimilation (Kendrick et al., 2017). All samples from Suiko, Koko, and the step heated values from Detroit have K/Cl similar to the range of previously measured contamination‐free MORB/OIB (Kendrick et al., 2012; Kendrick, Arculus, et al., 2014; Kendrick, Jackson, et al., 2014) samples, indicating that the Br/Cl and I/Cl enrichment is not a feature of seawater assimilation/contamination. Data from the crushing release of Detroit have K/Cl, Br/Cl, and I/Cl similar to samples, which contain an assimilated seawater component (Kendrick, Arculus, et al., 2013), indicating the fluid component of Detroit may contain an assimilated seawater component or has a very low abundance of lithophile K within the fluid inclusions. MORB = mid‐ocean ridge basalts; OIB = ocean island basalts; HIMU = high μ.

The Br/Cl and I/Cl ratios for each olivine analysis are displayed in Figure 3, along with previously determined values for MORB and OIB samples (Kendrick et al., 2012; Kendrick, Jackson, et al., 2014). The Br/Cl weight ratios obtained by crushing and heating range between 2.5 × 10^−3^ and 7.2 × 10^−3^, which overlaps with but extends to values more than twice the maximum measured MORB value (2.9 × 10^−3^) (Kendrick et al., 2012). The I/Cl weight values determined by both crushing and stepped heating show a similarly wide range, 3.1 × 10^−5^ to 34.7 × 10^−5^, and in common with Br/Cl, they overlap with and extend beyond the MORB mantle range (Figure 3). There are also discernible differences in I/Cl ratios for each seamount. Detroit has an average I/Cl ratio of 6.4 × 10^−5^ with several samples overlapping the range of I/Cl ratios measured in MORB glasses (Kendrick et al., 2012). The average I/Cl ratios of Suiko (14.7 × 10^−5^) and Koko (28.6 × 10^−5^) are higher and do not overlap with MORB values. It is also notable that the average K/Cl weight ratio from crushing and step heating of Detroit olivine is 14, within error of the MORB value (Kendrick et al., 2012). Average K/Cl values for Suiko and Koko are higher at 50 and 25, indicating an enrichment of lithophile K, relative to the more volatile Cl (Figure 4 and Table 2).

### 3.3 Noble Gases

**Figure 5:**
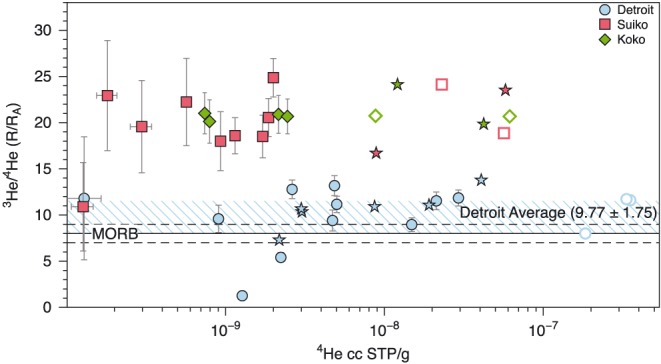
^3^He/^4^He versus ^4^He (log) concentrations (at standard temperature and pressure, STP) for individual crushing steps of the Emperor seamount olivines. Detroit olivines have consistently lower ^3^He/^4^He and higher ^4^He concentrations than the two younger seamounts. The ^3^He/^4^He of Detroit is within the range of values reported for MORB, while Suiko and Koko have high ^3^He/^4^He indicative of greater contribution from the primordial Hawaiian plume component. Open symbols represent the total gas release for each sample, and stars represent the previous analyses of the same samples from Keller et al. (2004). Average value of MORB (8 ± 1 R_A_; Graham, 2002) is displayed by the black dashed lines. MORB = mid‐ocean ridge basalts.

Helium concentrations and ^3^He/^4^He ratios from stepped crushing of all samples are displayed in Table 3 and Figure 5. The ^3^He/^4^He of the Detroit samples, calculated from summing all crushing steps, ranges between 8.0 and 11.7R_A_. The ^3^He/^4^He of the Suiko and Koko seamounts are consistently higher than Detroit, with the Suiko samples ranging from 18.9 to 21.1R_A_, and both Koko samples having ^3^He/^4^He of 20.7R_A_. The ^3^He/^4^He of all seamounts are similar to those previously obtained by Keller et al. (2004), although helium concentrations in Suiko and Koko measured during this study are lower on average (Figure 5). This may be due to variable efficiencies in the different crushing extraction techniques (e.g., multiple crushing steps and preheating of the samples, compared to single crushing step without preheating used by Keller et al. (2004)) or sample heterogeneity given the small number of previous measurements of the Suiko and Koko olivines.

**Table 3:** Total Helium Concentration and Isotopic Ratios From Combined Crushing Steps

Sample	Weight (g)	^4^He cm^3^/g STP (×10^−9^)	Error	^3^He/^4^He R/R_A_	Error
Detroit 50‐65	0.85	35.31	1.49	11.60	0.22
Detroit 43‐48	1.05	33.44	1.12	11.72	0.50
Detroit 100‐105	1.27	18.37	0.76	8.00	0.58
Suiko 27‐2	1.27	2.30	0.12	24.13	1.98
Suiko 24‐7	1.23	5.66	0.17	18.86	0.67
Koko 13‐18	1.30	0.88	0.05	20.74	3.38
Koko 10‐18	1.35	6.15	0.18	20.68	0.28

*Note*. Ratios are calculated taking the average of each crushing step. Uncertainties for ^3^He/^4^He are calculated by taking the standard error on the standard deviation of multiple crushing measurements. STP = standard temperature and pressure. Uncertainties are reported to 1*σ*.

Neon and argon concentrations and isotopic ratios are presented in the supporting information. All samples have ^20^Ne/^22^Ne ratios within error of air. The ^40^Ar/^36^Ar ratios of the olivines from all the seamounts are essentially atmospheric. Only one sample, Detroit 50–65, has an ^40^Ar/^36^Ar ratio (566 ± 24) significantly greater than the atmospheric value (298.6; Lee et al., 2006). The Suiko and Koko samples are enriched in the heavy noble gas elements with ^132^Xe/^36^Ar values that are above and ^22^Ne/^36^Ar below, the atmospheric and seawater values, respectively (Figure S6). The ^132^Xe/^36^Ar of the Detroit samples are also higher than seawater, but the ^22^Ne/^36^Ar is more similar to the mantle value suggesting that Detroit samples may have retained more mantle‐derived gases than Suiko and Koko. With the exception of He however, the noble gas signature of the Emperor seamount olivines appears to be dominated by an adsorbed atmospheric component.

## 4 Discussion

### 4.1 Origin of Emperor Seamount's Halogens: Source Vs. Contamination

The Br/Cl and I/Cl of the Suiko and Koko seamounts are distinct from Detroit and previously measured OIB samples (Figure 3). The elevated Br/Cl and I/Cl within the younger Emperor seamounts represents a significant departure from halogen signatures of other OIB and MORB glass samples, which have relatively consistent ratios, potentially suggesting that the mantle is more heterogeneous with respect to the halogens than has been previously suggested (Kendrick et al., 2017). In order to determine whether the halogen geochemical signatures within Emperor seamount olivine are indeed primary and representative of the mantle source, the efficacy of olivine phenocrysts to preserve mantle halogen signatures and resist overprinting from the surrounding marine environment must be assessed.

The halogen composition of the mantle has been routinely determined through the analysis of submarine basaltic glass (Kendrick, Jackson, et al., 2014; Kendrick et al., 2012, 2017). Chlorine, bromine, and iodine are all highly incompatible and exhibit no evidence of fractionation during partial melting or crystallization, indicating that halogens in basaltic glass samples are representative of the source composition (Kendrick et al., 2012). The direct incorporation of melt inclusions into olivine phenocrysts also enables the determination of the Cl composition of the mantle (Hauri, 2002). Chlorine within melt inclusions is considered to act as a closed system and is therefore not affected by diffusion‐driven loss (Bucholz et al., 2013), making them a good candidate for analyzing the halogen composition of the mantle when fresh basaltic glass is unavailable as is the case for the Emperor seamounts (Figure 1).

Previously, it has been shown that halogen signatures within submarine basaltic glass can be influenced by seawater assimilation (Broadley et al., 2017; Kendrick, Arculus, et al., 2013; Kendrick, Jackson, et al., 2014; Michael & Cornell, 1998) and, anomalously high I/Cl in glass has been attributed to alteration to palagonite (Kendrick et al., 2012). Seawater/brine assimilation into basalt melts and lavas normally produce lower than mantle K/Cl, from the assimilation of Cl‐rich seawater. This has been found to occur together with variations in Br/Cl and I/Cl, with submarine basalts exhibiting elevated Br/Cl and depleted I/Cl from the assimilation of brines that have undergone hydrothermal boiling and wall rock interaction within the magma chamber (Figure 4, Kendrick, Arculus, et al., 2013). In addition to seawater assimilation within the magma chamber and during eruption, submarine basalts are susceptible to alteration while residing within the marine environment (Chavrit et al., 2016). The Br/Cl of the Emperor seamount olivine is similar to the fluid component contained with altered oceanic crust (AOC) samples, potentially indicating that the samples have been overprinted by the introduction of circulating oceanic crustal fluids (Chavrit et al., 2016). However, the K/Cl, Br/Cl, and I/Cl of the Emperor seamounts from step heating analysis show no evidence of correlation, indicating that the assimilation of the seawater into the magma chamber prior to eruption or from alteration after eruption has been limited and is not responsible for the enriched Br/Cl and I/Cl values measured within the samples.

While there is no evidence of seawater assimilation within the fluid phase of the samples released during crushing of the Suiko and Koko samples, the samples from Detroit exhibit a similar K/Cl‐Br/Cl relationship to samples that contain an assimilated seawater component (Figure 4). The K/Cl of fluid inclusions, however, may not be representative of the source composition as volatile Cl can be concentrated in the fluid phase relative to lithophile K (Broadley et al., 2016), resulting in low K/Cl. The relationship between K/Cl and Br/Cl‐I/Cl in the crushed analysis of Detroit is based solely on two samples that have lower than mantle K/Cl, while retaining mantle‐like Br/Cl and I/Cl (Figure 4). The low K/Cl of the Detroit fluid inclusions may therefore be the result of low K concentrations within fluid inclusions rather than being evidence for seawater assimilation.

The noble gas signatures of the olivines provide a means to further evaluate the extent to which halogens are mantle derived or overprinted by alteration. Atmospheric noble gas in mantle‐derived samples can be introduced either by direct addition of atmospheric gases in the mantle source/magma chamber or from surficial contamination during interaction with air/seawater (Broadley et al., 2017; Farley & Craig, 1994). Evidence of atmospheric noble gases in the mantle source has been shown by the correlation between mantle ^3^He and atmospheric ^36^Ar within fluid inclusions of mantle xenoliths (Broadley et al., 2016; Matsumoto et al., 2001), although no such correlation exists in the Emperor seamounts indicating that the atmospheric component within the samples is a later addition (supporting information Figure S5). Olivine can irreversibly adsorb atmospheric noble gases with the degree of adsorption controlled by the surface area of the minerals (Protin et al., 2016). The ^22^Ne/^36^Ar and ^130^Xe/^36^Ar signatures (Figure S6) of the olivines appear to represent a fractionated seawater or atmospheric component, enriched in heavy noble gas elements from preferential adsorption of heavier noble gases onto the surface of the minerals during low temperature alteration in the oceanic crust (Chavrit et al., 2016; Podosek et al., 1981). While the adsorption of noble gases onto the surface of the olivines can account for the predominantly atmospheric noble gas signature, it remains unclear how this style of alteration could introduce marine halogens to olivine crystals. There is no statistical correlation between the amounts of potentially adsorbed seawater/atmospheric noble gas and the K/Cl, Br/Cl, and I/Cl of the Emperor seamount olivines (Figure S6). The process leading to the adsorption of noble gases on to the surface of the olivine crystals therefore did not result in the introduction of halogens from the surrounding marine environment.

Finally, no clear correlation exists between halogen signatures and the degree of alteration within the samples, with all samples being altered to a similar degree (Regelous et al., 2003; supporting information). Careful consideration was taken during this study to avoid samples exhibiting any evidence of alteration, and all samples underwent rigorous cleaning in nitric acid and acetone to remove any surface alteration. The halogen signature of the samples are not consistent with evidence of assimilation and/or alteration present in other samples (Chavrit et al., 2016), and degassing of the halogens is assumed to be limited given the submarine eruption of the samples; we therefore suggest that given all the available evidence, the halogen signatures within the Emperor seamount appear to be related to the original melt composition. However, low halogen concentrations within the olivines and the evidence for limited surficial alteration contend that halogens introduction during alteration cannot be completely ruled out.

### 4.2 Evolution of the Emperor Seamount Mantle Source

The evolution of the Br/Cl and I/Cl from MORB‐like values in Detroit toward more elevated values in Suiko and Koko occurs concurrently with changes in other geochemical tracers (Keller et al., 2004; Regelous et al., 2003). Detroit basalts have ^3^He/^4^He and ^87^Sr/^86^Sr at the low end of the range of values for young Hawaiian lavas (Kurz et al., 1983) and similar to the range of MORB values (Graham, 2002; White et al., 1987), whereas the Suiko and Koko basalts have higher ^3^He/^4^He and ^87^Sr/^86^Sr, more typical of the modern Hawaiian Islands (Keller et al., 2004; Regelous et al., 2003). The mantle source of the Emperor seamounts therefore appears to have evolved from a more MORB‐like composition during the eruption of Detroit seamount to a composition more akin to the modern Hawaiian Island prior to the formation of the Suiko and Koko seamounts.

The MORB‐like geochemical signature within Detroit has been attributed to greater amounts of depleted upper mantle being entrained into the plume. It has been suggested that this may be the result of the Hawaiian hot spot being in close proximity to a spreading ridge during the formation of Detroit in the Late Cretaceous (Keller et al., 2000; Regelous et al., 2003). The close proximity of the plume to a spreading ridge (Emperor fracture zone) could have resulted in the entrainment of depleted mantle material into the plume (Hekinian et al., 1999). However, recent analyses of the Emperor fracture zone concluded that it most likely represents an oceanic transform fault and not an extinct spreading ridge (MacLeod et al., 2017). Melting of the plume at shallower depths and pressures, under younger (<10 Ma) thinner lithosphere (Caplan‐Auerbach et al., 2000), is another possible mechanism for the entrainment of depleted upper mantle material into the Detroit seamount. The depth and pressure within the mantle can directly control the melting point of different mantle lithologies, which in turn can impart different geochemical signatures on resulting magmatic products (Niu et al., 2011; Sobolev et al., 2005, 2007).

If changes in lithospheric thickness and therefore changes in the dominant mantle lithology contributing to the Emperor seamounts are the causes of the geochemical evolution, then this should be evident in changes in trace element signature of the different seamounts. Detroit olivines have somewhat elevated Ni compositions compared to MORB at a given Fo but have lower Ni content at similar Fo numbers when compared to other OIB and the Suiko and Koko seamounts. This indicates that there was enrichment in Ni within the mantle source between the formation of Detroit and the younger Suiko and Koko seamounts (Figure 2; Niu et al., 2011; Sobolev et al., 2005, 2007). Calcium and Mn concentrations of Suiko and Koko olivines are also lower than would be predicted for olivines crystallizing from a melt derived from the MORB mantle source (Sobolev et al., 2007; Walter, 1998).

Melting within olivine‐free pyroxenite, formed from the reaction of subducted eclogite and mantle peridotite, has been suggested to produce Ni‐enriched olivine within intraplate volcanic settings (Sobolev et al., 2007). Subducted oceanic crust present as eclogite in the mantle is presumed to melt at greater depths and pressure than the surrounding depleted mantle peridotite. These eclogite melts react with the surrounding peridotite to form olivine‐free pyroxenite, which upon further melting can ultimately result in an enriched Ni and depleted Mg, Ca, and Mn signature (Suiko and Koko) compared with peridotite‐derived melts (Detroit). Similar high Ni, low Ca, and Mn signatures may also result from peridotite melting at high temperature and pressures in the mantle as the bulk partition coefficient of Ni between olivine and melt ( ${K_{d}}_{\mathrm{Ni}}^{\mathrm{Ol}/\text{melt}}$) decreases with increasing pressure. Therefore, melt formed at higher pressure will be enriched in Ni (Niu et al., 2011; Putirka et al., 2011). However, the Ni enrichment in the modern Hawaiian Islands is considered to more likely result from changes in pyroxenite contributions rather than to high temperature and pressure melting of peridotite, as similar temperature and pressures are predicted for the eruption of the Iceland plume on Baffin Island and West Greenland yet the Ni content within the West Greenland olivines is ~1,000 ppm lower (Herzberg et al., 2013).

The amount of recycled crust present as pyroxenite in the Hawaiian plume mantle source has been estimated to be 15–20% (Sobolev et al., 2007). The amount of pyroxenite melt that contributes to the seamounts can be calculated using the Ni and Mg concentration of the olivines following the procedure of Sobolev et al. (2005). The seamounts show a progressive enrichment in pyroxenite‐derived melt, with Detroit, Suiko, and Koko samples containing a minimum pyroxenite melt contribution of 46%, 58%, and 70%, respectively. The estimates for Detroit are consistent with previous predictions for the Detroit seamount (~40%), while Suiko and Koko are similar to the values reported for the current Hawaiian Islands (~60%; Sobolev et al., 2007).

**Figure 6:**
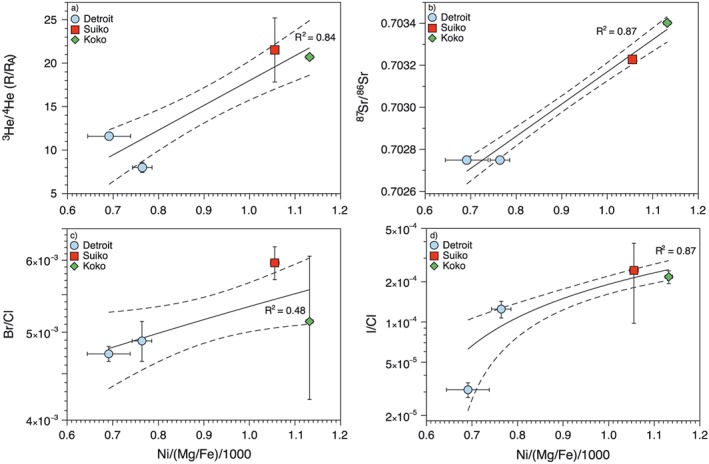
Concentration of Ni relative to Fo number [Ni/(Mg/Fe)/1000] versus ^3^He/^4^He (a), ^87^Sr/^86^Sr (b), Br/Cl (c), and log I/Cl (d) of the Emperor seamounts. All ratios show a positive correlation with increasing Ni/(Mg/Fe) taken as a proxy for pyroxenite contribution. The pyroxenite component is considered an integral component to the plume source indicating that the high ^87^Sr/^86^Sr, Br/Cl, and I/Cl originated as part of the pyroxenite component within the high ^3^He/^4^He plume. The Suiko and Koko seamounts therefore have a higher proportion of plume material (including subducted pyroxenite) relative to Detroit, which has a higher contribution from depleted mantle peridotite (see text). The ^3^He/^4^He, Br/Cl, and I/Cl represent the average and standard deviation (1*σ*) of all analyses of the samples, while the ^87^Sr/^87^Sr is from Keller et al. (2004). Black lines represent the line of best fit through the data, while the dashed lines are the 1*σ* confidence intervals.

The Ni/Fo ratio (taken as a proxy for pyroxenite contribution) exhibits a positive correlation with the Br/Cl, I/Cl, ^3^He/^4^He, and ^87^Sr/^86^Sr ratios in the three Emperor seamounts (Figure 6). This suggests that the subduction‐derived pyroxenite component is integral to the plume, with the high ^3^He/^4^He and ^87^Sr/^86^Sr from the Hawaiian plume mantle source and the high Br/Cl and I/Cl from the subducted pyroxenite manifesting together in the younger seamounts when melting occurs at high pressures. There is no significant change in the ^3^He/^4^He, Br/Cl, and I/Cl between the eruption of Suiko and Koko despite continually increasing levels of pyroxenite contribution. The ^87^Sr/^86^Sr ratio however continues to increase between Suiko and Koko (Figure 6). Why the Sr continues to evolve while the He and halogen ratios stabilize is unknown. It may be related to the volatile elements being more sensitive to the input of plume material. As such they may become dominated by the plume component at much lower contributions relative to the more lithophile elements including Sr (Agranier et al., 2005).

The correlation between pyroxenite contributions, Br/Cl, I/Cl, and ^3^He/^4^He indicates that subducted material has been incorporated into the primordial undegassed Hawaiian mantle plume source. Dehydrated slab material and mantle metasomatized from the release of slab fluids are envisaged to reach the deep mantle (Dixon et al., 2017). The higher than MORB K/Cl ratio of Suiko and Koko is the result of the incorporation of volatile (Cl)‐depleted subducted material into the Hawaiian plume, as suggested for other plume mantle sources (Kendrick, Jackson, et al., 2014). The subduction of oceanic crust and metasomatized mantle into the deep primitive mantle may therefore result in the incorporation of recycled oceanic crust in the form of pyroxenite into buoyant mantle plumes and may therefore account for the coupled high ^3^He/^4^He and subduction‐derived geochemical signature of the Hawaiian plume (Figure 6).

The evolving geochemical signature of the Emperor seamounts appears directly influenced by the overlying lithospheric thickness during partial melting. Lithostatic pressure ultimately arrests the melting beneath oceanic hot spots, as a result this controls the melting of distinct lithologic components within the ascending mantle if their solidus temperatures differ (Ito & Mahoney, 2005). The thinner lithosphere and decreased lithostatic pressure that prevailed during the formation of Detroit seamount led to incorporation of a higher proportion of peridotite‐derived melt. Melting of this peridotite would have introduced shallow‐depleted mantle volatile, which explains the MORB‐like ^3^He/^4^He and trace element signature of Detroit seamount when compared to Suiko, Koko, and the modern Hawaiian Islands. The increasing lithospheric thickness with time led to a change from predominantly peridotite‐derived melts (Detroit seamount) to melts containing a larger proportion of pyroxenite within the plume. The geochemical signature within Suiko and Koko seamounts therefore has not been diluted to the same degree as that at Detroit seamount, where the incorporation of depleted mantle melts has significantly obscured the geochemical signatures originating from other mantle components within the plume.

### 4.3 Identifying the Subducted Components Within the Emperor Seamounts

A pyroxenite signature originating from subducted material within the Hawaiian plume source based on the trace element signature of the Emperor seamounts should also be apparent in the radiogenic and volatile element signatures. The Sr‐Nd‐Hf‐Pb isotopic composition of the Hawaiian Islands indicates that the Hawaiian plume is geochemically heterogeneous. The Emperor seamount chain appears to only contain the Kea component with no evidence of the Loa‐like component being present prior to the eruption of the Kauai island in the Hawaiian Island chain (Tanaka & Nakamura, 2005). The Kea component is characterized by low ^87^Sr/^86^Sr and high ^143^Nd/^144^Nd, ^176^Hf/^177^Hf and radiogenic Pb isotopic ratios, interpreted to represent the presence of a section of the lower oceanic crust within the plume introduced either through the assimilation of the oceanic lithosphere during melt transit or from direct addition of recycled oceanic crust or melts thereof to the Hawaiian plume mantle source (Dixon et al., 2017; Eiler et al., 1996; Hanano et al., 2010; Lassiter & Hauri, 1998; Tanaka & Nakamura, 2005).

The similarity of the Br/Cl and I/Cl within the three Emperor seamounts to that of potential subducting volatile reservoirs, including AOC and serpentinites, may also suggest that volatile elements such as the halogens have been introduced into the plume through subduction recycling (Chavrit et al., 2016; Svensen et al., 2001, Figure 3). Samples from the Hawaiian Islands thought to sample the Kea component have δ^18^O and δ^11^B similar to that of hydrothermally AOC or oceanic lithosphere, further suggesting the Kea component contains an AOC volatile component (Eiler et al., 1996; Tanaka & Nakamura, 2005). Hawaiian olivines retain constant δ^11^B while exhibiting variations in B concentrations and radiogenic isotopic ratios, indicating that the variable degrees of oceanic crust assimilation during melt transit cannot explain the B isotopic signature of the Kea component (Tanaka & Nakamura, 2005). Furthermore, the radiogenic Os isotopes within Hawaiian lavas require up to 75% oceanic crust assimilation that is considered unlikely given the high MgO, Ni, and ^3^He/^4^He primitive nature of the samples (Lassiter et al., 2000). The source of the AOC‐like signature in Kea is therefore most likely related to the introduction of oceanic crust to the Emperor seamount mantle source (Tanaka & Nakamura, 2005).

While the Emperor seamounts appear to have a subducted oceanic crust‐like component within the mantle source, the origin and mechanism for delivery of these AOC‐like volatiles is most likely related to the subduction of serpentinites (Kendrick et al., 2011; Kobayashi et al., 2017; Pagé & Hattori, 2017; Scambelluri et al., 2004). This is because serpentinites can be strongly enriched in fluid mobile elements such as halogens and boron as well having a large stability field, therefore allowing the subduction of marine volatiles deep (200 km) into the mantle (Kendrick et al., 2011; Schmidt & Poli, 1998). Serpentines can also retain the isotopic and elemental signature of the circulating fluids that are driving the serpentinization, as indicated by seafloor serpentinites having high I/Cl indicating the involvement of marine pore fluids during their formation (Kendrick et al., 2011; Kendrick, Arculus, et al., 2013; Kendrick, Honda, et al., 2013).

During serpentinite subduction, Cl is lost preferentially compared to other volatile such as B, which is retained throughout subduction and be recycled back to the deep mantle (Tanaka & Nakamura, 2005). Despite the preferential loss of Cl during subduction there remains an appreciable amount of Cl within subducting serpentinites even beyond the antigorite stability field, highlighting the potential for halogens to be subducted deep in to the mantle. Up until the breakdown of antigorite, the Br/Cl and I/Cl of subducting serpentinites remain similar to their seafloor counterparts (Kendrick, Honda, et al., 2013; Pagé & Hattori, 2017). After the final breakdown of antigorite to chlorite and the subsequent transformation of antigorite serpentinite to chlorite harzburgite however, both the Br/Cl and I/Cl ratios fall to values lower than the range of MORB and OIB samples (Kendrick et al., 2018). In order for the mantle to retain its relatively homogenous Br/Cl and I/Cl signature the bulk subducting slab must have a halogen composition similar to the mantle (Kendrick et al., 2018) or Br and I are preferentially released and isolated in subcontinental lithospheric mantle (Broadley, Barry, et al., 2018; Broadley, Kagi, et al., 2018). Garnet peridotites, which represent the final dehydration product of chlorite harzburgites, have been shown to have higher than mantle Br/Cl (Figure 3) and may therefore be the high Br/Cl reservoir needed to maintain a homogenous mantle halogen composition (Kendrick et al., 2018). The higher than MORB K/Cl ratio of Suiko and Koko is the result of the incorporation of volatile (Cl)‐depleted subducted material into the Hawaiian plume (Kendrick, Jackson, et al., 2014). The subduction of oceanic crust and metasomatized mantle into the deep primitive mantle may therefore result in the incorporation of both components into buoyant mantle plumes and as such can account for the coupled high ^3^He/^4^He and subduction‐derived geochemical signature of the Hawaiian plume (Figure 6).

The progressive dehydration of the subducting slab results in a wide variability in the Br/Cl and I/Cl of the subducting lithologies relative to their starting composition. Br/Cl and I/Cl may therefore not be applicable for tracing the subduction of surface volatiles such as sediments and AOC beyond the breakdown of antigorite within the slab as it appears dehydration and fractionation play a fundamental role in controlling halogen compositions. The final dehydration of chlorite harzburgite leads to the loss of Cl from the slab and leaves the resulting garnet peridotite with higher Br/Cl and I/Cl (Hughes et al., 2018; Kendrick et al., 2018). The similarities in halogen composition between subducted garnet peridotites and the Suiko and Koko seamounts indicate that a dehydrated section of oceanic crust may be the source of the higher than mantle Br/Cl and I/Cl within the Hawaiian‐Emperor seamount mantle source. As discussed, the higher Br/Cl and I/Cl in Suiko and Koko occur concurrently with other elemental and isotopic tracers indicating the mantle source contains an oceanic crust component. While the halogens within the Emperor seamount mantle source may not be directly related to the subduction of AOC fluids, the variety of data indicating the presence of an oceanic crustal component within the mantle source (Lassiter et al., 2000; Tanaka & Nakamura, 2005) suggests that the halogens are also derived from this subducted component (Figure 3).

While the process of dehydration may result in the subducting slabs on the whole having similar halogen compositions to previously determined mantle samples, the possible identification of halogen signatures within the Emperor seamounts outside the mantle range indicates that, in common with other volatile species such as O and S (Cabral et al., 2013; Eiler et al., 1996), the mantle may retain subduction‐derived halogen heterogeneities. Why these heterogeneities have not been identified in other OIB, which are interpreted to contain a greater proportion of recycled oceanic crust, is unknown (Kendrick et al., 2015, 2017). Samples from the Mangaia OIB have the most extreme ^238^U/^204^Pb signature, which defines the high μ (HIMU) end‐member; however, they appear to be dominated by a peridotite mantle source rather than a recycled pyroxenite source as in the Emperor seamounts (Herzberg et al., 2014). Furthermore, Pitcairn seamount has Br/Cl and I/Cl within the defined mantle range with similar Sr and Pb isotopes to the Hawaiian mantle source (Kendrick, Jackson, et al., 2014), but it contains a significantly lower proportion of recycled material (5%, Delavault et al., 2015) within the mantle source compared to that calculated for the Hawaiian mantle source (15–20%, Sobolev et al., 2007). Therefore, there appears to be an apparent decoupling between the isotopic signatures and the source lithology. The high Br/Cl and I/Cl in the Suiko and Koko seamounts may therefore be the result of a combination of a higher proportion of younger recycled material (<1.5 Ga; Eisele et al., 2003) in the mantle source that has better resisted homogenization, together with a high‐pressure melting domain. Halogens within previously measured OIB including the Detroit seamount may therefore have been more easily overprinted by the larger contribution of peridotite in the source or during low‐pressure melting and are therefore more representative of the average convecting mantle signature. Further coupled trace element and halogen analysis within other OIB, including those with well‐preserved basaltic glass, could further test whether variations in pyroxenite contribution control the geochemical signature of OIB as seems to be the case for the Emperor seamounts.

## 5 Conclusion

The geochemical signature of the Emperor seamount chain has evolved from having a primarily MORB‐like volatile and trace element signature toward signatures similar to the younger Hawaiian Islands. From 65 Ma (Suiko seamount) and younger, olivines in Emperor seamount basalts are more enriched in Ni and more depleted in Ca and Mn, while retaining more primitive ^3^He/^4^He. This change resulted from increased melting depth beneath the Pacific lithosphere as it aged, leading to a larger contribution by melting of recycled pyroxenite within the plume source when it was beneath the younger seamounts. Thinner lithosphere and lower lithostatic pressure during melt generation beneath the oldest seamount (Detroit) at 76 Ma led to a stronger component of melting of depleted peridotite that was accompanied by MORB‐like mantle ^3^He/^4^He signatures.

Elevated halogen ratios of Suiko and Koko samples are similar to the garnet peridotite products of serpentinite dehydration and may signal the subduction of oceanic crust into the Hawaiian plume mantle source. The mantle is therefore not completely homogenized with respect to subducted halogens and noble gases as previously considered. Evidence for distinct recycled components in the mantle may however be readily overprinted by ambient mantle peridotite in OIB with a smaller recycled crust component or when melting occurs at lower pressures in the mantle such as in the Detroit seamount.

## Supporting information



Supporting Information S1

Data Set S1
