# Supplementary material for: Recycled Components in Mantle Plumes Deduced From Variations in Halogens (Cl, Br, and I), Trace Elements, and 3He/4He Along the Hawaiian‐Emperor Seamount Chain
Source: Geochem Geophys Geosyst. 2019 Jan 14;20(1):277–94. doi: 10.1029/2018GC007959 (PMC6472562; doi:10.1029/2018GC007959)
Supplement: Supplementary file 1 — Supporting Information S1 [file GGGE-20-277-s001.pdf]

Geochemistry, Geophysics, Geosystems

Supporting Information for

Recycled components in mantle plumes deduced from variations in halogens (Cl, Br, I), trace elements and $^{3}He/^{4}He$ along the Hawaiian- Emperor Seamount chain

Michael W. Broadley[1,2*]; Hirochika Sumino[3]; David W. Graham[4]; Ray Burgess[1]; Chris J. Ballentine[5]

[1]School of Earth and Environmental Sciences, University of Manchester, Oxford Road, Manchester M13 9PL, UK

[2]Centre de Recherches Pétrographiques et Géochimiques, 54501 Vandoeuvre-Lès-Nancy Cedex, France

[3]Department of Basic Science, Graduate School of Arts and Sciences, The University of Tokyo, Tokyo 153-8902, Japan

[4]College of Earth, Ocean, and Atmospheric Sciences, Oregon State University, Corvallis, Oregon, USA

[5]Department of Earth Sciences, University of Oxford, South Parks Road, Oxford, OX1 3AN, UK

Contents of this file

Figure S1. Microscope image of Detroit thin section.
Figure S2. Microscope image of Suiko thin section.
Figure S3. Microscope image of Koko thin section.
Figure S4. Backscatter electron (BSE) image of Detroit, Suiko and Koko olivine phenocrysts.
Figure S5. Plot of ^{3}He concentrations vs. ^{36}Ar concentrations.
Figure S6. Halogen elemental ratios vs. $^{22}Ne/^{36}Ar$ and $^{132}Xe/^{36}Ar$.

Table S1. Raw noble gas data from individual crushing steps
Table S2. Elemental data for each point measurement using Electron Microprobe Analysis (EMPA)

Introduction

This supplementary section contains a series of petrographic images, figures and raw data. Figures S1-3 show the mineralogy and level of alteration within each of the Emperor Seamounts. Figure S4 includes a BSE image of olivine phenocrysts representative of each seamount. Figure S5 plots the concentration of ^{3}He vs ^{36}Ar, indicating that there is no correlation between mantle He and atmospheric Ar indicating that atmospheric contamination is not inherent to the mantle source but was most likely added during or after

formation. Figure S6 compares the halogen and noble gas elemental ratios to determine whether the samples have been contaminated from the surrounding marine environment.

Table S1 presents all the He, Ne and Ar concentrations and isotopic ratios measured during each crushing step. Tables S2, presents the elemental data from each individual spot measurement from the electron microprobe.

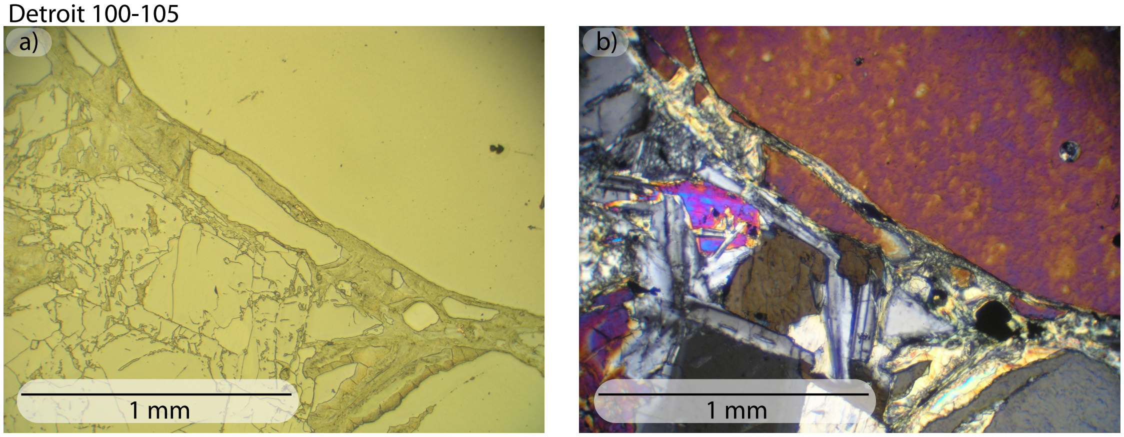

Figure S1. a) Reflective microscope and b) cross-polarized, images showing general mineralogical composition of Detroit Seamounts samples. The samples contain large olivine phenocrysts (~3 mm) within a predominantly plagioclaise matrix, with minor amounts of clinopyroxene and devitrified glass. The sample shows no evidence of veins with secondary minerals (quartz, clay etc.) indicating fluid flow through the sample was minimal. The sample is generally free from extensive alteration with olivine alteration to iddingsite being minor and limited to the rims of the phenocryst.

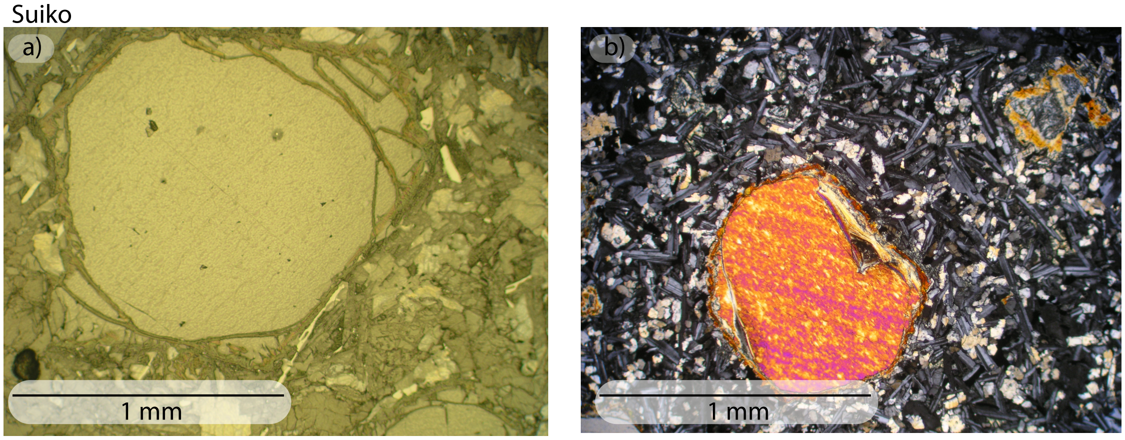

Figure S2. a) Reflective microscope and b) cross-polarized, images showing general mineralogical composition of Suiko Seamounts samples. Olivine phenoscryst are slightly smaller (~1 mm) compared to Detroit and Koko. The plagioclase-dominated groundmass

is very fresh with little evidence of alteration. Alteration is limited to the formation of iddingsite along the rims of olivine phenocrysts and the devitrification of glass (image b).

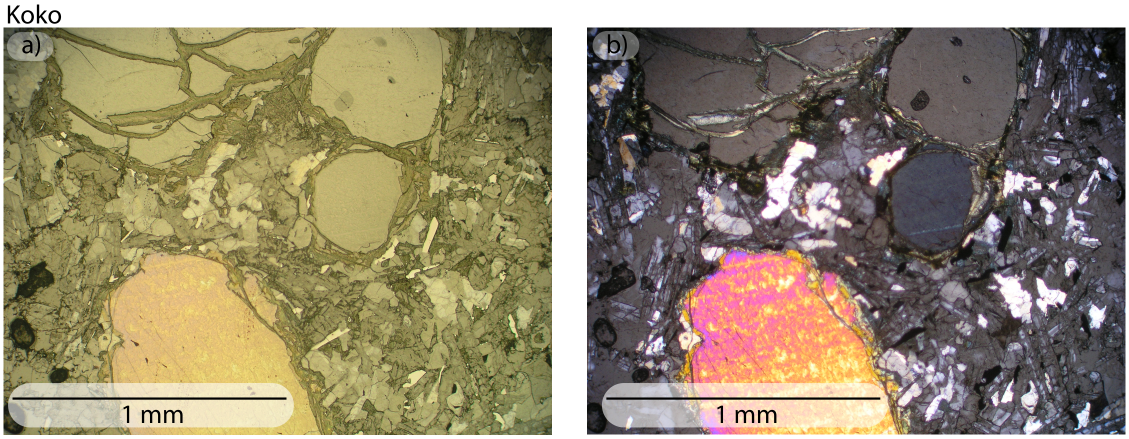

Figure S3. a) Reflective microscope and b) cross-polarized, images showing general mineralogical composition of Koko Seamounts samples. The sample also contain large olivine phenocrysts (~2 mm) within a predominantly plagioclaise matrix, with minor amounts of clinopyroxene and devitrified glass. Alteration is limited with some alteration to clay and iddingsite around the rims and along fractures of the olivines.

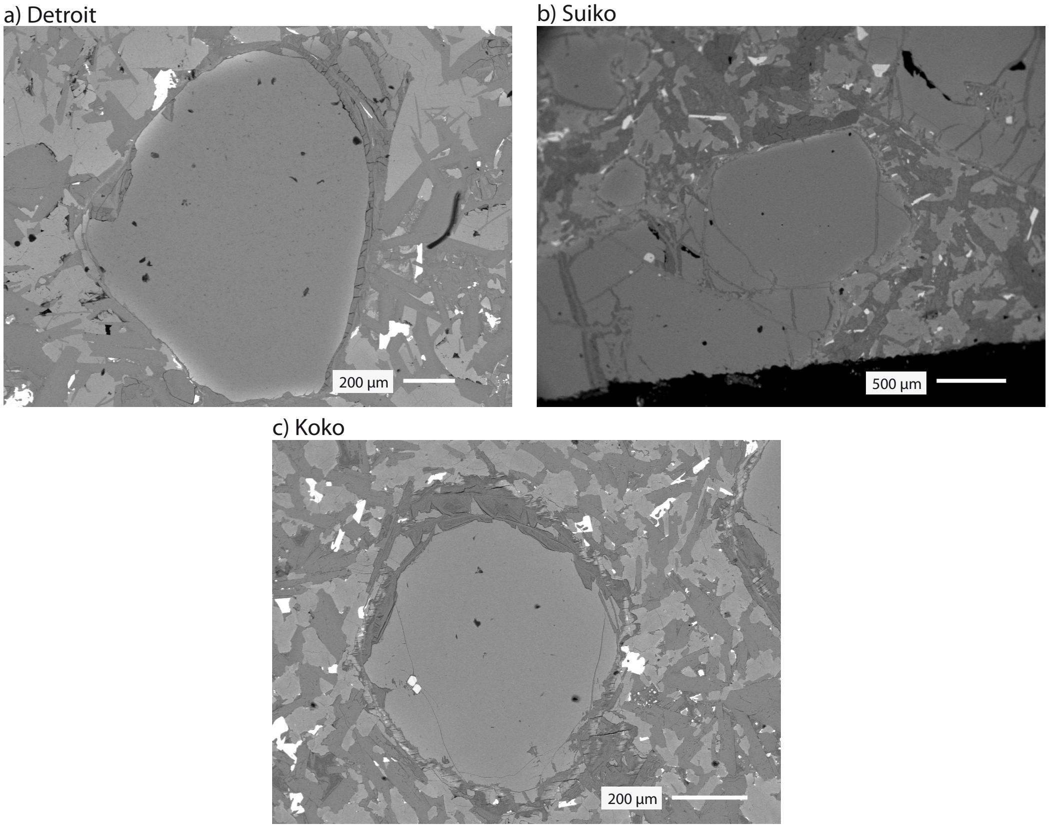

Figure S4. Backscatter electron (BSE) image of Detroit (a), Suiko (b) and Koko (c) olivine phenocrysts. BSE images highlight the fresh nature of the olivine phenocrysts. Crystals edges are well defined in Detroit and Suiko indicating alteration is limited, whilst Koko exhibits minor alteration to iddingsite around the crystal rims.

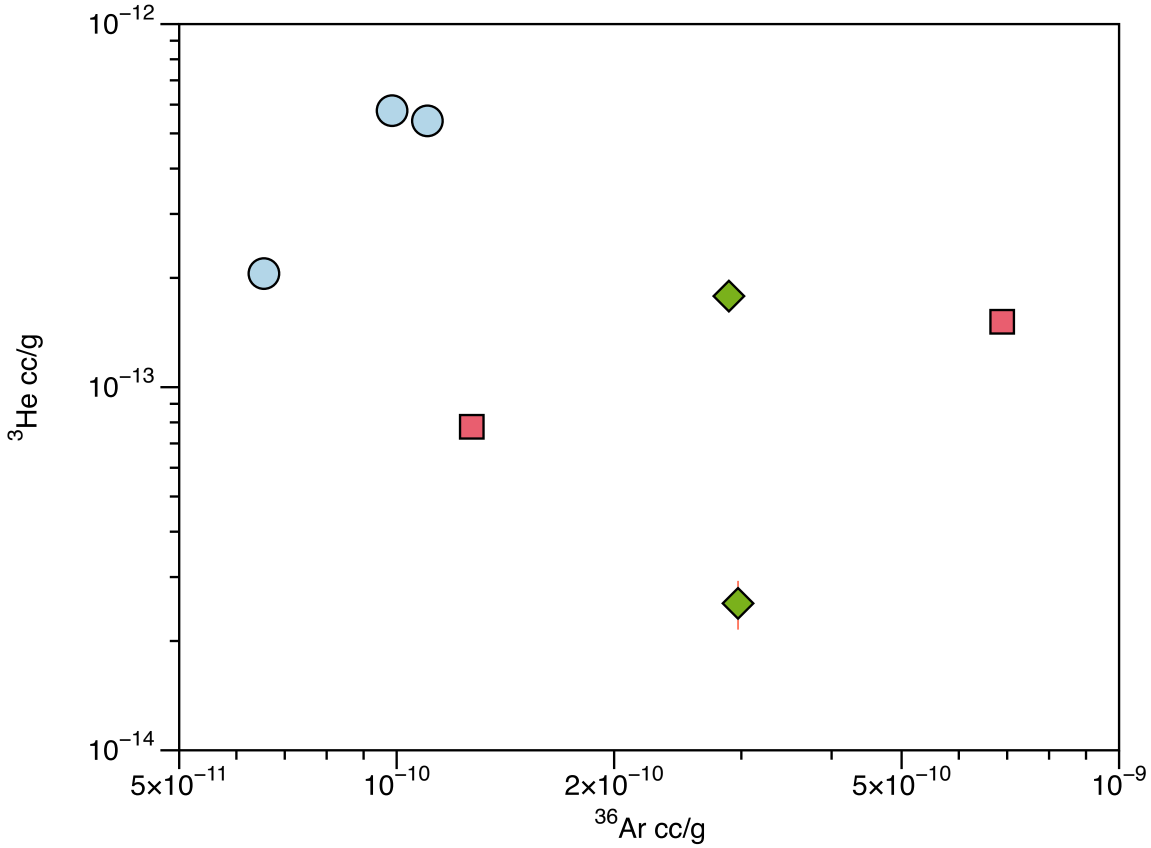

Figure S5. Concentrations of ^{3}He vs. ^{36}Ar released during crushing. With the exception of $^{3}He/^{4}He$, all noble gas isotopic ratios are atmospheric suggesting that the original mantle signature has been over-printed by atmospheric contamination (Supplementary Information). Atmospheric noble gas in mantle-derived samples can be introduced either by direct addition of atmospheric gases in the mantle source / magma chamber, or from surficial contamination during interaction with air/seawater (Broadley et al., 2017; Farley and Craig, 1994). Evidence for the introduction of atmospheric noble gases to the mantle source has been shown by using a correlation between mantle ^{3}He and atmospheric ^{36}Ar within fluid inclusions of mantle xenoliths (Broadley et al., 2016; Matsumoto et al., 2001). There is no clear correlation between mantle derived ^{3}He and atmospheric ^{36}Ar indicating that the addition of atmospheric noble gases to the samples did not occur within the mantle source of magma chamber where a positive correlation would be expected (Broadley et al., 2016). Atmospheric contamination of the samples most likely occurred during eruption or continuously whilst residing in the oceanic crust.

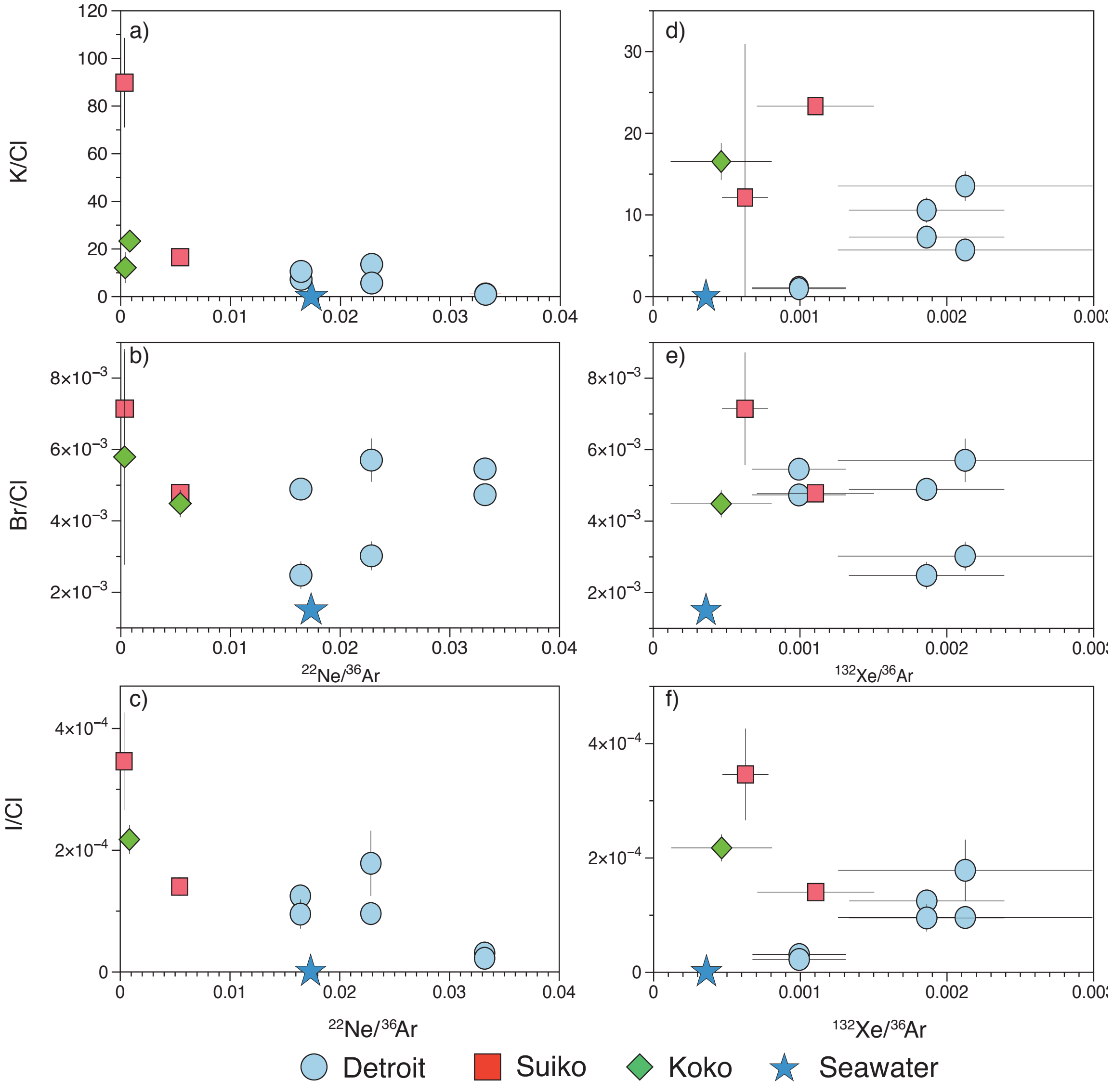

Figure S6. Relationship between halogen and noble gas elemental ratios. All olivines have $^{130}Xe/^{36}Ar$ greater than seawater, indicating the samples have been enriched in heavy elements possibly during alteration on the seafloor. The $^{22}Ne/^{36}Ar$ of the Detroit samples are however greater than seawater towards the average mantle values suggesting they may still retain a magmatic volatile component. There is no statistical significant correlation between the $^{22}Ne/^{36}Ar$, $^{130}Xe/^{36}Ar$ and the K/Cl, Br/Cl and I/Cl of the samples indicating that the process responsible for introducing the atmospheric component to the samples did not introduce any seawater derived halogens (blue star).
